# Pediatric isoniazid-resistant tuberculosis of the bone marrow manifesting as hemophagocytic syndrome: A case report

**DOI:** 10.3389/fped.2022.1051414

**Published:** 2022-11-21

**Authors:** Junfeng Zheng, Yongbin Xu, Jun Yang, Ke Cao, Guofang Deng, Peize Zhang

**Affiliations:** ^1^ Department of Pulmonary Medicine and Tuberculosis, The Third People's Hospital of Shenzhen, Shenzhen, Guangdong, China; ^2^ Department of Immunology, Shenzhen Children's Hospital, Shenzhen, China; ^3^ Department of Clinical Laboratory, Shenzhen Children's Hospital, Shenzhen, China

**Keywords:** isoniazid-resistant, tuberculosis, bone marrow tuberculosis, hemophagocytic syndrome, child

## Abstract

Hemophagocytic syndrome (HPS) is a critical syndrome of ineffective hyperinflammatory immune response resulting in infiltration of lymphocytes and histiocytes in various organs. Causes can be hereditary or due to malignancy, autoimmune disease, or infection. HPS due to *Mycobacterium tuberculosis* is rare as only a handful of cases are reported, and they are mostly associated with severe disseminated tuberculosis (TB). We reported a 9-year-old boy with tuberculosis of the bone marrow accompanied with hemophagocytic syndrome. The patient presented with manifestation of HPS and had no respiratory symptoms or risk factors for TB but was later diagnosed of isoniazid-resistant TB in the bone marrow. He had a good outcome after receiving anti-TB drugs and corticosteroids on time. This case highlights that bone marrow might be a shelter for *Mycobacterium tuberculosis*. Concurrent testing for drug susceptibility in TB cases with an uncommon manifestation is recommended even for first episodes. Early diagnosis and etiological confirmation of the infection origin and appropriate treatment are essential to improve survival in this otherwise life-threatening condition.

## Introduction

Childhood tuberculosis (TB) is still a challenging health problem around the world. It is estimated that around 1.1 million children every year are infected with *Mycobacterium tuberculosis* globally. Infectious disease, including TB is among the leading causes of global childhood morbidity and mortality (1, 2). Hemophagocytic syndrome (HPS), also known as hemophagocytic lymphohistiocytosis (HLH), is a critical syndrome of hyperinflammatory response characterized by excessive activation of macrophages, leading to the aberrant release of cytokines and phagocytosis of blood cells. HLH can be caused by primary or secondary immune abnormalities (3). Primary HPS is genetic and can be confirmed by gene sequencing. Secondary HPS is often caused by infection and malignancy (4). Treatment of HLH aims at controlling hypercytokinemia and eliminating the infected cells. *M. tuberculosis* have been reported to be one of the triggering factors for secondary HPS. Most reported HPS cases were in adults with severe disseminated TB whereas HPS in children with drug-resistant TB was rarely reported.

Here, we report a case of TB of the bone marrow accompanied with HPS in a child. The patient presented with the onset of HPS and had no respiratory symptoms or risk factors for TB but was later diagnosed of TB in the bone marrow, with isoniazid resistance. He had a good treatment outcome after receiving anti-TB drugs and corticosteroids.

## Case description

A 9-year-old boy with intermittent fever for 2 months was transferred to our hospital on November 3, 2021. He was born with alpha thalassemia (-SEA/*α*α), and from his birth to 2018, he had received five red blood cell transfusions. He has been Bacilli Calmette–Guerin (BCG) vaccinated at birth under the national vaccination program. He has no recent travel history, and his parents reported no family hereditary diseases, besides thalassemia, no TB history nor TB exposure.

The patient started to develop intermittent fever in August 2021, with the highest body temperature of 38.0°C and night sweats. There were no respiratory symptoms or weight loss. He was admitted to a local children hospital shortly after his fever had persisted for 5 days. His routine blood test on admission showed white blood cell (WBC) 2.6 × 10^9^/L, neutrophils (Neu) 1.15 × 10^9^/L, hemoglobin (Hb) 74 g/L, and platelets (PLT) 211 × 10^9^/L. Ultrasound scan revealed bilateral cervical lymphadenopathy and an enlarged spleen.

**Figure 1:**
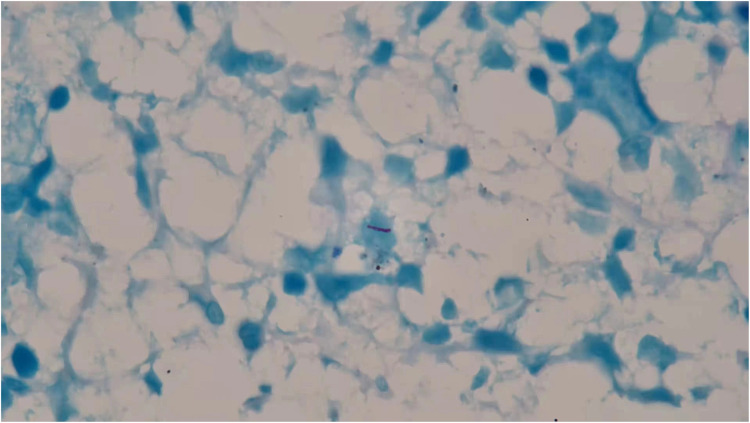
*Mycobacteria tuberculosis* identified in bone marrow aspirate smear.

In 3 days, his hemoglobin level dropped to 64 g/L, and he was given 2U red blood cell infusion. Repeated blood test on the following day showed decreased level of WBC to 1.5 × 10^9^/L and Neu of 0.51 × 10^9^/L with an elevated Hb level of 90 g/L and an unremarkable change of PLT 201 × 10^9^/L. Viral markers for Epstein–Barr virus (EB DNA) was negative. In addition, subsequent blood interferon-gamma release assays (IGRAs) and skin purified protein derivative (PPD) tests were positive. Lung computed tomography (CT) showed calcification of the right hilum and mediastinal lymph nodes. In the absence of lung infiltration and tuberculous foci, latent tuberculosis infection (LTBI) was suspected. A bone marrow aspiration smear performed the following day showed active bone marrow hyperplasia, significantly decreased neutrophil level, and generally normal morphology. The patient had persistent daily fever between 37.4°C and 38.8°C accompanied with dry cough, fatigue, and loss of appetite. He was empirically administered with amoxicillin 0.25 g three times a day for 3 days and antifebrile for a week. But his fever remained unsolved during the treatment, fluctuating in the same range as before. Pathological findings from his cervical lymph node biopsy showed no caseous necrosis and no acid-fast mycobacteria. The persistent low WBC in peripheral blood had raised concern. To look into possible causes of the persistent fever, a second bone marrow puncture was performed. Bone marrow smear was positive for acid-fast staining (Figure 1). Disseminated TB infection was suspected. But further sputum and cerebrospinal fluid smear were negative for both acid-fast staining and TB-DNA (CapitalBio Technology, China). Thus, pulmonary TB and tuberculous meningitis were ruled out. The bone marrow was presumed as the tuberculous origin. Anti-TB regimen composing isoniazid, rifampicin, pyrazinamide, and ethambutol was initiated from the diagnosis. His temperature resumed to normal range 4 days later. His appetite also improved.

**Figure 2:**
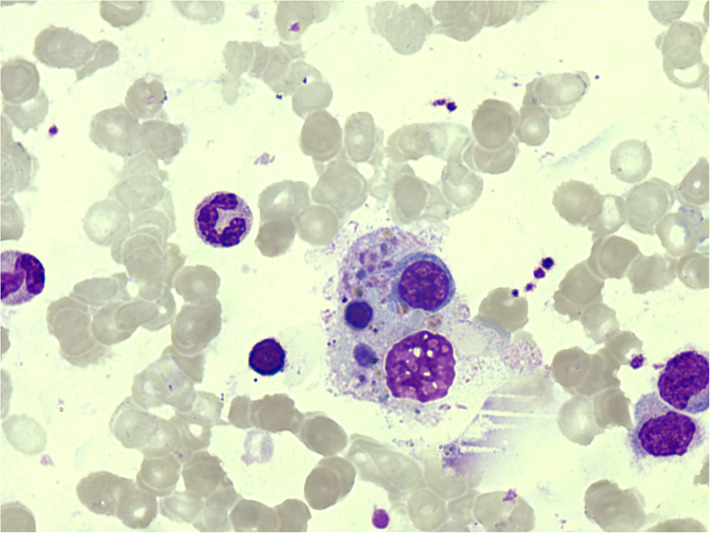
Hemophagocytic cells found in bone marrow examination.

But a week later, he had fever again with the highest measure of 40.0°C. Serum ferritin was observed to have elevated to 3,603.35 ng/ml (reference: < 300 ng/ml). Methylprednisolone 1 mg/kg q12 h was administered as anti-inflammatory therapy, but his fever did not go away. A repeated bone marrow aspiration was performed the following day to investigate the causes for the unresolved fever, elevated serum ferritin, and decreased WBC. Bone marrow biopsy showed active hyperplasia with occasional hemophagocytic histiocytes, mainly phagocytizing white blood cells, red blood cells, and platelets (Figure 2). Bone marrow for real-time PCR (qPCR) assay for TB was positive; moreover, gene sequencing revealed the promotor region of InhA gene mutation (−15C > T), which caused resistance to isoniazid. Bone marrow for TB culture was performed but had finally turned out negative. Serum ferritin was precisely monitored and was found to further elevated to 9612.81 ng/ml in 5 days. HPS was confirmed, and 10 mg/kg of methylprednisolone was prescribed daily. In 2 days, the patient's fever resolved. Serum ferritin dropped to 5,755.54 ng/ml and WBC in peripheral blood was restored to a normal level. Whole-genome sequencing confirmed no variants of exon. This test result rule out the known primary genetic causes of HLH. Diagnosis was confirmed as having (1) bone marrow TB with isoniazid resistance; (2) secondary HPS.

Before administration of isoniazid-resistant TB regimen modification, the patient developed nausea, loss of appetite without fever 18 days after initial anti-TB regimen. Systematic examination revealed yellow skin and sclera with hepatosplenomegaly. Liver function test showed total bilirubin (TBIL) of 111.1 μmol/L, directed bilirubin (DBIL) 67.3 μmol/L, alanine transaminase (ALT) 520.5 U/L, and aspartate transaminase (AST) 321.5 U/L, which were consistent with drug-induced liver injury (DILI). He was transferred to our hospital for specialized tuberculosis management where his regimen was reviewed and modified to the following: linezolid 300 mg/day, ethambutol 0.5 g/day, and levofloxacin 0.25 g/day based on his body weight of 25 kg. Methylprednisolone was reduced to 2 mg/kg/q8h. As serum ferritin was gradually decreasing and his body temperature and WBC were normal, methylprednisolone was lowered to 0.4 mg/kg/q12h 12 days later.

**Figure 3:**
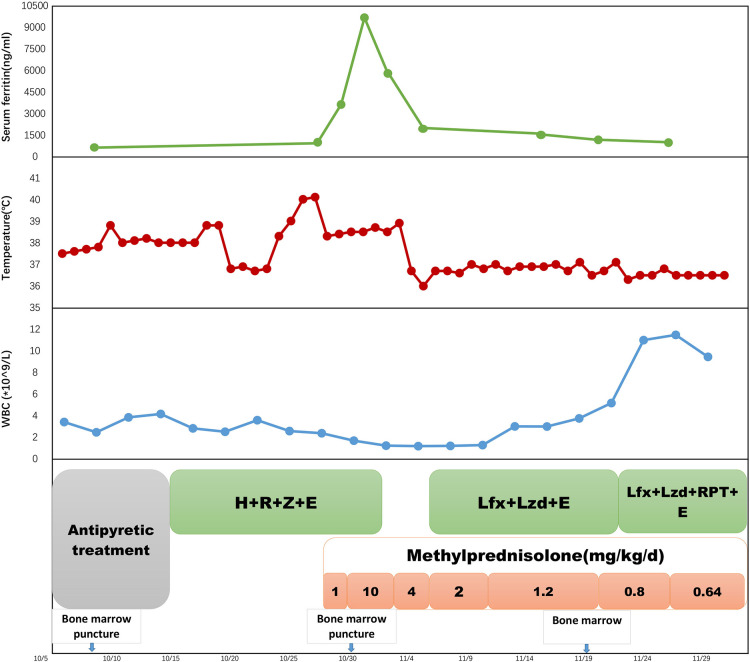
A timeline of treatment and clinical events with laboratory parameters. H, isoniazid; R, rifampicin; Z, pyrazinamide; E, ethambutol; Lzd, linezolid; Lfx, levofloxacin. RPT, rifapentine.

After 15 days of the above therapy, a follow-up evaluation of bone marrow aspirate for metagenomic next-generation sequencing (mNGS) and qPCR assay showed no detection of *M. tuberculosis*. Subsequent laboratory investigations confirmed normal liver function and a lowered serum ferritin level of 1,150.0 ng/ml. A 0.3-g rifapentine was added twice a week to his anti-TB regimen. He was discharged with the prescribed methylprednisolone at a tapering dose of 4 mg every 10 days (Figure 3) until the next follow-up consultation. He is being followed up in our outpatient unit for the next 2 years for this rare medical condition. The child has no more fever, but his hepatosplenomegaly persisted. He has returned to school and is leading a normal life. As of press time, the patient's anti-TB treatment is still ongoing.

## Discussion

HPS is a histiocyte disorder characterized by the activation and proliferation of macrophages leading to the uncontrolled phagocytosis of platelets, erythrocytes, and lymphocytes and their hematopoietic precursors throughout the reticuloendothelial system (5). Secondary HPS can occur at any age and is mostly associated with malignancy, autoimmune disease, drug hypersensitivity, or infection. Viruses, especially Epstein–Barr, is the most common pathogens of infection-associated HPS (6). *M. tuberculosis*, as a facultative intracellular pathogen, also causes HPS. TB-related HPS occurred mostly in patients with severe disseminated TB. Most of them were in critical situation at the time of HPS onset. Some patients required mechanical ventilation and extracorporeal membrane oxygenation (ECMO) support and prolonged hospital stay for survival (7–10).

The disease onset of our patient presented persistent fever with decreased level of WBC and Hb. The decrease in Hb was confounded with his hereditary disease, and splenomegaly could also relate to the fact of him being a blood recipient, but the persistent fever of unknown origin and the progressive drop of WBC suggests that another underlying cause may be responsible. After primary HPS was ruled out by exon sequencing, we speculated that the cause maybe non-hereditary. In the early stage of the disease, lung CT only showed calcification of the right hilar and mediastinal lymph nodes with no remarkable manifestations relating to disseminated TB. Even though IGRAs was positive, it did not provide adequate evidential support to confirm the diagnosis of active TB. Because of the lack of reasonable grounds, determining whether HPS was associated with mediastinal lymph node TB at this timepoint was difficult. The investigation for possible risk factors for HPS narrowed when we detected acid-fast bacterium in the bone marrow. We thus considered that the HPS was associated with bone marrow TB. Molecular biology analysis further confirmed the bacilli strain to be isoniazid resistant. Current studies have shown that bone marrow mesenchymal cells provide a good shelter for *M. tuberculosis* and lead to persistent infection (11). Therefore, we speculated that the occurrence of HPS was a cause of tuberculous infection at the bone marrow. However, it was difficult to confirm how TB infection in the bone marrow induced over activation and proliferation of the macrophage and lymphocyte and led to HPS in this child.

There is scarce literatures worldwide regarding the epidemiology of the incidence of TB of the bone marrow. In a retrospective study in an area with high TB incidence in China, only 14 out of 110 patients with bone marrow granulomas tested culture positive for acid-fast bacilli or TB-DNA qPCR, which are microbiological evidence of TB (12). This demonstrated that TB of the bone marrow is difficult to diagnose except for some severe disseminated cases which manifested specific TB clinical characteristics. Furthermore, no drug-resistant TB of bone marrow had been reported to date. To our knowledge, this is the first case that presented with isoniazid-resistant TB of the bone marrow without disseminated TB but manifested as HPS. Noteworthily, it suggests that TB-associated HPS not only occurs as a complication in patients with severe disseminated TB, rather TB infection of the bone marrow may be a trigger itself to the development of HPS.

All reported TB-related HPS cases emphasized the importance of timely diagnosis and initiation of anti-TB treatment. Mortality rate in patients with delayed treatment has been reported to be as high as 50% in previous literatures (13). However, with effective anti-TB therapy and immune treatment for HPS, most patients reported positive treatment outcome with no relapse. After confirmed diagnosis of TB, the pediatric patient in our case received effective anti-TB treatment and steroid for his HPS. Even though he has transient DILI, his symptoms resolved, and *M. tuberculosis* was cleared in his bone marrow after treatment. His HPS was completely relieved after the infection was controlled. The good treatment outcome also demonstrated the importance of etiological investigation in patients with HPS caused by infection.

In conclusion, this case reminds us that bone marrow TB and drug resistance should be considered in HPS patients, especially in countries with a high prevalence. Pathological and etiological examination of the bone marrow can provide clues for diagnosis. Medical institutions with adequate resources are recommended to perform drug susceptibility testing in patients confirmed with TB, even it may be the first episode. Early diagnosis and etiological confirmation of the infection origin and appropriate treatment are essential to improve survival in this otherwise life-threatening condition.

## Data Availability

The raw data supporting the conclusions of this article will be made available by the authors, without undue reservation.
